# Quantitative Analysis of Postural Instability in Patients with Parkinson's Disease

**DOI:** 10.1155/2021/5681870

**Published:** 2021-04-13

**Authors:** Yang Yu, Siquan Liang, Yue Wang, Yanan Zhao, Jiaojiao Zhao, Haitao Li, Jingchao Wu, Yuanyuan Cheng, Fan Wu, Jialing Wu

**Affiliations:** ^1^Department of Neurological Rehabilitation, Tianjin Huanhu Hosptial, Tianjin, China; ^2^Department of Neurosurgery, Tianjin Huanhu Hosptial, Tianjin, China; ^3^Department of Neurology, Tianjin Huanhu Hosptial, Tianjin, China

## Abstract

**Introduction:**

Postural instability is commonly observed in Parkinson's disease, leading to an increasing risk of falling and worsening as the disease progresses. We found that limit of stability can be applied to reflect the dynamic evolution of postural instability in patients with Parkinson's disease.

**Methods:**

Forty-three patients (9 of Hoehn and Yahr stage I, 12 of stage II, 14 of stage III, and 8 of stage IV) met the criteria for the diagnosis of idiopathic Parkinson's disease and could stand independently for at least 10 minutes. Twelve healthy controls with no sign of parkinsonism were also recruited. Postural instability was assessed by posturography in different directions (forward, backward, right, left, forward-right, forward-left, backward-right, and backward-left). This study trial was registered with the Chinese Clinical Trial Registry (no. ChiCTR1900022715).

**Results:**

All participants were able to complete the limit of stability tasks without any complications. Patients in stages II to IV exhibited smaller end point excursion and slower time to complete than controls, suggesting an impaired limit of stability. The patients in stage II exhibited a remarkable decline in most directions compared to controls, except for right and left, and forward and backward decline occurred the earliest. For patients in stage III, right was the only direction with no significant difference from controls. In stage IV patients, the limit of stability declined significantly in all directions (*p* < 0.05).

**Conclusions:**

The postural abnormalities of Parkinson's disease can occur at early stages, and the pattern of decline is more severe in the forward-backward direction. This trial is registered with ChiCTR1900022715.

## 1. Introduction

Parkinson's disease (PD) is a progressive and chronic neurodegenerative disorder. The main clinical manifestations include bradykinesia, tremor, rigidity, and gait/postural disturbance. Postural instability (PI) is commonly observed in patients with PD, leading to an increased risk of falling, which has a negative impact on the patient's ability to perform daily activities [1, 2]. Like dysphagia, autonomic dysfunction, and cognitive impairment, postural instability is also one of the most important disease milestones in advanced Parkinson's disease, especially represented by the transition to Hoehn and Yahr (H&Y) staging 2 to 3 [3]. Notably, due to the progressive nature of PD, these symptoms tend to gradually worsen over time. Therefore, early identification of PI in individuals with PD is important for further prevention. However, patients do not tend to consult the doctor unless PI is severe with frequent falls. From the standpoint of clinical, PI in PD is only noticed in middle and late stages (Hoehn and Yahr (H&Y) stages III–V), the phases in which significant disability generally occurs [4].

The assessment of PI in PD patients is the “pull test” according to the Movement Disorders Systems-Unified PD Rating Scale (MDS-UPDRS) item 3.12. This test requires the examiner to pull on the patient's shoulder from behind and catch them while they start to fall [5]. However, this test cannot provide reliable information regarding the stability of the patient in daily life, and it does not reflect the variety of situations in which a fall may occur except for the backward postural reaction in the context of a mechanical perturbation under static conditions. The Berg Balance Scale (BBS) is another widely used evaluation method; it is a subjective evaluation method that can be influenced by many unpredictable factors. Also, the BBS is limited to proactively identify underlying impairments in postural stability, and these measures have a documented ceiling effect in the early to middle stages of PD [4, 6, 7]. Thus, the measures are rater-dependent, nonmetric, and lack objectivity. In view of the present situation, objective evaluation is needed to identify PI characteristics accurately so as to enable clinical individualization.

The limit of stability (LOS) can objectively reflect posture stability in ambulatory PD patients by posturography [5]. LOS assesses voluntary postural control by measuring the individual's active limits of stability, quantifying movement excursion [4, 7]. Furthermore, LOS presents the interlimb coordination based on different task requirements in different directions, and the increase in LOS scores suggests a good control of balance toward the specific directions.

The purpose of this paper is to study the availability of LOS in quantitatively analyzing PI of PD, especially during the early stages. We had three objectives: to determine the stage of PD when LOS decline occurs, to clarify the direction in which LOS is more likely to descend, and to explore the specific patterns of PI in PD patients. On this basis, we aim to provide a sensitive means for measuring PI deficits in clinical settings and select a targeted treatment plan according to the objective quantitative posturographic analysis for future research and clinical application.

## 2. Methods

### 2.1. Subjects

All patients met the 2015 MDS criteria for the diagnosis of idiopathic PD and exhibited definite responses to dopaminergic medication [8]. Forty-three PD patients who were matched for age, gender, weight, and height were recruited from the outpatient clinic of Tianjin Huanhu Hospital and received stable PD medication for the last 2 months. All of the patients could stand independently for at least 10 minutes. Exclusion criteria included the following: unable to stand independently as determined by H&Y stage V; diagnosis of atypical parkinsonism (e.g., multiple system atrophy, progressive supranuclear palsy, corticobasal degeneration) and secondary parkinsonism (vascular parkinsonism, normal pressure hydrocephalus); comorbidities that may affect posture and balance, including the history of other neurological diagnoses, peripheral neuropathy, impaired proprioception, vestibular disorders, visual disturbances, and musculoskeletal disorder in the back or lower limbs within the last 3 months that limits independent standing; diagnosis of dementia; or inability to speak/understand Chinese.

PD patients were classified based on H&Y staging score (*n* = 9, stage I; *n* = 12 stage II; *n* = 14, stage III; and *n* = 8, stage IV). Twelve healthy controls (HCs) with no sign of parkinsonism and matched for the same parameters were also recruited.

This trial was registered in the Chinese Clinical Trial Registry (http://www.chictr.org.cn; no. ChiCTR1900022715). The procedures were approved by the Tianjin Huanhu Hospital ethics committee (no. 2019–35). All participants provided written informed consent before entering the study.

### 2.2. Experimental Protocol

Motor dysfunction rating was based on the Movement Disorder Society-Unified Parkinson's Disease Rating Scale motor examination (MDS-UPDRS III) [7], which was administered by the principal investigator, an expert trained and certified by the International Parkinson's and Movement Disorders Society. PI testing was used to document the LOS assessed by posturography (TecnoBody, PROKIN Systems, Italy). The LOS assesses voluntary postural control, requiring the individual to move his or her center of mass (COM) to eight different directional targets. Variables measured in this study were average endpoint excursion (EPE) and time to complete the test (TCT). EPE is defined as the displacement of the COM during the primary attempt toward the designated target, expressed as a percentage of the maximum LOS. TCT is defined as the time from the presentation of a start cue to the completion of all test tasks in the voluntary shifting of the participant's COM toward the target position [7, 9]. The tests were administered by one qualified rater and standardized testing procedures and instructions followed as described below. All subjects were assessed in the morning, at least 12 h after the last dose of antiparkinsonian medications in order to reduce the effect of dopaminergic medications on posturographic findings.

To assess the LOS, the subjects were asked to stand in a neutral position with their feet shoulder width apart on a circular platform. They were asked to shift their COM to reach a maximal distance in the target direction as quickly and accurately as possible without moving their feet until they felt like they must break their base of support to prevent themselves from falling. The eight targets were spaced at 45° intervals around the COM and represented on a computer monitor, including forward (FW), backward (BW), right (RT), left (LT), forward-right (FW-RT), forward-left (FW-LT), backward-right (BW-RT), and backward-left (BW-LT) [6, 10]. All LOS measurements were performed in a supervised manner. All subjects completed two test trials, with approximately 20 seconds rest between the trials. Participants were allowed to have one practice trial before each test trial [11].

### 2.3. Statistical Analysis

We compared baseline demographics and clinical characteristics of each group. Descriptive statistics are presented as the mean ± standard deviation. For all variables, the normality of the data distribution was assessed by the Shapiro–Wilk statistic. The data difference in each group was evaluated by one-way analysis of variance (ANOVA) with a two-sample *t*-test of the Kruskal–Wallis rank sum test and Mann–Whitney test for post hoc pairwise tests for continuous and ordinal variables. Bonferroni's adjustment was used for multiple comparisons between groups. To compare the data on LOS between H&Y stages and HCs, one-way ANOVA or the Kruskal–Wallis rank sum test was performed with the two-sample *t*-test or Mann–Whitney tests for post hoc pairwise comparisons.

We also tested the association between the LOS and the patients' clinical features, including age, sex, weight, height, MDS-UPDRS, LEDD, and the association between the TCT and the clinical features using Pearson correlations.

All statistical analyses were performed in SPSS software (version 22.0.0.1; IBM, Inc., USA). The level of significance was set to *p* < 0.05.

## 3. Results

### 3.1. Demographics

The clinical characteristics of the patients are shown in Table 1. Age, gender, weight, and height were not significantly different between H&Y stages I to IV and HCs. Not unexpectedly, PD patients at different stages had significantly different equivalent daily dosages of levodopa (*p* < 0.05). H&Y PD patients of stages III and IV had significantly longer disease duration (*p* < 0.05) and higher MDS-UPDRS (III) score (*p* < 0.05).

### 3.2. LOS Parameters

**Figure 1:**
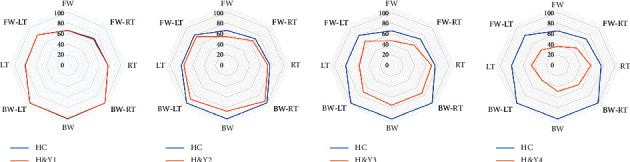
Directional analysis of limit of stability. H&*Y*=Hoehn–Yahr stage, HC = healthy control, FW = forward, BW = backward, RT = right, LT = left, FW-RT = forward-right, FW = forward-left, BWRT = backward-right, BW-LT = backward-left. ^*∗*^*p* < 0.05, ^*∗∗*^*p* < 0.01.

All participants were able to complete LOS tasks without any complications. None of the patients underwent any change in medication during the study period. The results are shown in Table 2 and Figure 1. The deficits in LOS parameters were likely disease stage-dependent, as H&Y stage I patients were not significantly different from HCs. In contrast, the H&Y patients of stages II to IV had significantly smaller EPE and slower TCT than the HC group, suggesting impaired LOS.

### 3.3. Directional Analysis

The directional analysis of LOS is shown in Table 2 and Figure 1. We found no significant difference between the patients at H&Y stage I and HCs in any of the eight directions, and the patients in H&Y stage II exhibited a remarkable decline in most directions, except for RT (*p*=0.733) and LT (*p*=0.488), compared to HCs. For H&Y stage III patients, RT (*p*=0.105) was the only direction that was not significantly different from HCs. The LOS of H&Y stage IV PD patients clearly declined significantly in all directions.

Figure 1 offers a clear picture of LOS parameters. The decline in LOS occurred earlier in FW and BW directions (H&Y stage II) than RT and LT directions. Furthermore, the PD patients in H&Y stage II had a greater decline in the BW (15.04 ± 12.78) than FW direction (13.12 ± 7.77), suggesting that PD patients maintained greater stability in the FW direction than the BW direction, regardless of the asymmetry of motor signs, but it has not reached significance (*p*=0.479).

## 4. Discussion

The aim of this study was to quantitatively analyze PI in PD patients, especially in the early stages of PD. First, the PD patients had smaller EPE and slower TCT, even as early as H&Y stage II. Second, directionwise analysis indicated that BW and FW stability were more easily influenced in PD patients than RT and LT. Third, early-stage PD patients exhibited a greater decline in the BW direction than the FW direction, which suggests that PD patients maintained greater stability in the FW direction regardless of the asymmetry of motor signs.

Most previous studies have focused on the advanced stages of the disease; when the pull test was significantly abnormal, the phenomenon of fall or near-fall had appeared, and dyskinesia was increased [9, 12]. Unfortunately, neither medication nor surgical treatment and rehabilitation is satisfactory at this period. In clinical practice, if we are able to find the underlying tendency for near-fall or fall earlier and keep the consistency of clinical balance tests, which are important for comprehensive management of patients with PD, subsequently, early detection of this tendency alerts physicians and enables early interventions to be put in place to reduce the negative impact [5, 13].

Our study results showed that the postural control system of the patients was affected already at an early stage of PD and that even H&Y stage II patients demonstrated a significant decline in LOS compared to HCs. This finding will help in customizing earlier rehabilitative measures for patients, aiming for an optimal control of balance and fall prevention.

Recently, Tuanzhi Chen et al. reported that when diverting attention or increasing cognitive task difficulty, the postural control system will easily be affected at an early stage of PD compared to HCs [14]. This study suggested the presence of underlying postural abnormalities in the early stages of PD. Thus, as shown in our study, deficits of PI may be commonly observed in early PD patients.

Mohan Ganesan et al. reported that the history of falls or near-falls and lost balance in PD patients does not necessarily correlate with the results of the pull test [15]. We also need prospective studies on falls in PD patients in the future and not only cross-sectional ones. Consistent with our findings, even the patients who do not complain of falls and who have a normal pull test (H&Y stage II) have smaller EPE (*p*=0.008) and slower TCT (*p*=0.021) than HCs. This further illustrates that the pull test is not enough for detecting PI at the early stages of PD [9, 14]. However, quantitative analysis of LOS parameters by posturography may provide a sensitive means of measuring the deficits of postural stability in a clinical setting.

The question of whether PD patients should be tested in the “OFF” or “ON” period arises. PD patients in an ON levodopa medication period will exhibit increasing postural involuntary sway, which may affect the postural stability [16, 17]. In addition, falls among PD patients occur more frequently in the OFF levodopa medication period. This uncertainty prompted us to investigate PI in PD patients during the OFF period.

LOS parameters declined gradually with disease progression and trunk rigidity gradually worsened. Horak et al. considered that the reduced postural stability margin was due to a slower increase and smaller peak center of pressure in the PD patients than in control subjects [18]. We think that one reason may be the greater involuntary “sway” area in all directions for PD in static posturography compared to HCs [5, 17]. This “sway” was defined as involuntary movement of the body either FW and BW or to the RT and LT side [13, 14]. One of the mechanisms of adverse compensation in involuntary sway requires trunk stiffness to reduce degrees of “sway” necessary for controlling posture stability [17, 19]. Lack of trunk flexibility could be influenced by the disease itself; due to widespread degeneration in PD involving the basal ganglia and its efferent connections, the capacity for change in conditions and coordination in trunk control should be worse in PD patients than HCs [18]. Therefore, axial rigidity and poor trunk coordination were exhibited in PD patients. The slower velocity of COM displacement compared to HCs is likely also due to that increased passive stiffness. This would explain why the LOS parameters decline with disease progression.

The results also suggest that the anterior-posterior directions are more prone to problems than LT-RT movement for LOS in PD patients. When testing LT-RT, their feet were separated, and the center of gravity displacements reciprocates motion between the two feet, making the force greater in the LT-RT directions than anterior-posterior directions [20, 21]. Therefore, lower levels of active trunk muscle activation are required, and more trunk flexibility was retained relative to the anterior-posterior directions [22].

In contrast, Mohan Ganesan et al. reported that the LOS scores of PD patients did not significantly differ from HCs at H&Y stage II and only started to show from H&Y stage III. However, this is consistent with our study showing a greater decline in the anterior-posterior directions than LT-RT at H&Y stage II [15]. Two possible factors can explain the discrepancy. First, the pull test is carried out in an artificial situation in which the patient is prepared and warned. Deciding on the H&Y stage relies on the presence of axial manifestations, which we need to bear in mind [22, 23]. Second, the subjects were evaluated in the “medication-on” state with posturography, and we do it in the “OFF” period.

On the basis of quantitative analysis, we performed an analysis basing on the directional analysis. The results suggesting that patients are more vulnerable to losing balance in the BW direction. This observation is probably due to the ankle and hip not exerting as much torque in the BW direction as in the FW (small dorsiflexor compared to plantar flexor) [24, 25]. The basal ganglia system normally compensates for this difference and produces relatively higher dorsiflexor muscle activation [26, 27]. This capacity was damaged in PD patients and worsened with disease progression. Individuals should also rely more on their visual function to maintain FW posture stability. As previous studies have shown, low illumination or lack of visual inputs is a potential exacerbator of PI in both older people and PD patients [4, 28]. The study by Frenklach and colleagues indicated that people with PD are unable to use the impaired proprioceptive feedback on a dynamic moving surface for orientation and that they have to rely on visual and vestibular feedback [12]. In contexts in which vision is absent, the BW direction for LOS would decline earlier in PD patients.

Recently, a few studies have attempted to perform objective posturographic assessments to determine specific patterns of PI [29, 30]. The current study investigated PD patients exhibiting PI as early as H&Y stage II, and the BW and FW directions of LOS were more easily influenced. Furthermore, PD patients maintained greater stability in the FW direction than the BW direction, regardless of the asymmetry of motor signs. Based on these findings, we speculate that the direction-specific impairment may be an initial pattern of PI in PD. When the impairment pattern progresses, it will be a formative factor of camptocormia as a kind of protective posture response. When the impairment is further generalized, it could manifest a tendency to near-fall or fall and result in an impaired pull test.

The current study has several limitations. First, the sample size was moderate with relatively small numbers of PD patients at every H&Y stage. The observations of direction-specific balance impairment still need to be validated in larger studies. Second, recent literature has reported that orthostatic hypotension (OH) affects ambulatory capacity in patients with Parkinson's disease, as well as in the prevalence of falls [31]. But the LOS that this study discussed is just the core range of active movement when patients are standing; it is a part of the balance system. Postural transitions and dynamic balance are not involved. Therefore, the influence of neurological orthostatic hypotension on falls was not considered, nor was it specifically analyzed in the paper. We will evaluate and discuss these issues when further research work about the sit-stand transition, dynamic balance, and other aspects is carried out. Third, EMG techniques will be used for future study to obtain information based on kinematics analysis to examine spatiotemporal changes during balance testing. A lack of subtype evaluation (e.g., PIGD and TD) could also limit further identification of the posturographic features of each subtype. Last, this analysis might be costly and time-consuming and require dedicated personnel. These characteristics may limit its wide application.

## 5. Conclusion

In general, the pathophysiology and neurobehavioral manifestation of PI in PD patients are remarkably complex. Our study identified postural abnormalities in the early stages of the disease and a pattern of direction-specific decreases in postural stability. The information is potentially useful for clinical practice to assist neurologists in the clinical differentiation and enable specific early guidance for goal-based intervention.

## Figures and Tables

**Table 1:** Participant demographics.

Group (*N* = 55)	H&Y I (*N* = 9)	H&Y II (*N* = 12)	H&Y III (*N* = 14)	H&Y IV (*N* = 8)	HC (*N* = 12)
Age (years)	59.67 ± 7.02	61.50 ± 8.37	65.21 ± 7.50	63.25 ± 8.96	62.90 ± 7.39
Gender (% female)	8.11	24.32	27.03	10.81	13.51
Weight (kg)	79.33 ± 10.07	71.07 ± 8.38	72.17 ± 12.28	70.43 ± 15.45	77.00 ± 11.38
Height (cm)	171.00 ± 3.61	168.93 ± 5.18	165.92 ± 6.40	166.14 ± 8.28	170.30 ± 5.79
MDS-UPDRS (III)	22.33 ± 1.80	33.75 ± 7.21	39.86 ± 1201	46.50 ± 11.33	
R	3.33 ± 1.00	5.50 ± 2.28	5.57 ± 2.21	6.75 ± 3.06	
B	10.00 ± 2.00	14.14 ± 2.88	16.38 ± 5.19	18.71 ± 7.06	
A	6.33 ± 2.18	8.33 ± 2.74	11.50 ± 1.09	13.75 ± 5.97	
T	4.00 ± 4.58	4.67 ± 3.34	11.36 ± 3.89	11.88 ± 1.13	
LEDD (mg)	166.67 ± 115.47	355.36 ± 142.16	447.88 ± 123.97	573.40 ± 162.65	
Disease duration (years)	1.67 ± 0.58	3.86 ± 2.14	5.85 ± 2.15	6.29 ± 1.38	

Data are presented as the mean ± standard deviation or proportion. H&*Y*= Hoehn and Yahr stage, HC = healthy control, MDS-UPDRS = Movement Disorder Society Revised Unified Parkinson's Disease Rating Scale, *R* = rigidity subscore, *B* = bradykinesia subscore, *A* = axial subscore, *T* = tremor subscore, LEDD = levodopa equivalent daily dose.

**Table 2:** Limit of stability results.

Group	HCs	H&Y I	H&Y II	H&Y III	H&Y IV	*p* value			
						I vs. HCs	II vs. HCs	III vs. HCs	IV vs. HCs
Time to complete test (s)	73.90 ± 2.96	78.67 ± 4.44	83.58 ± 10.54	82.21 ± 13.02	94.50 ± 9.77	0.280	0.021	0.040	0.000
Endpoint excursion (%)	84.22 ± 4.69	74.15 ± 12.42	64.91 ± 19.66	59.50 ± 20.72	50.42 ± 15.14	0.188	0.008	0.001	0.000
FW	65.63 ± 1.37	65.20 ± 0.74	53.88 ± 7.35	46.92 ± 15.34	36.24 ± 15.78	0.803	0.013	0.000	0.000
FW-RT	70.67 ± 0.89	68.71 ± 1.98	65.24 ± 4.29	55.11 ± 3.21	47.11 ± 10.35	0.383	0.012	0.000	0.000
RT	75.87 ± 2.97	75.77 ± 1.93	72.92 ± 12.29	69.61 ± 14.50	57.47 ± 4.87	0.906	0.733	0.105	0.012
BW-RT	99.92 ± 0.33	99.90 ± 0.10	95.25 ± 5.09	73.98 ± 7.06	51.36 ± 7.51	0.978	0.044	0.000	0.000
BW	100.53 ± 0.66	99.95 ± 0.10	86.61 ± 14.30	74.87 ± 8.52	48.98 ± 17.61	0.901	0.002	0.000	0.000
BW-LT	100.51 ± 1.09	100.56 ± 0.51	90.57 ± 6.35	70.19 ± 16.74	38.71 ± 5.99	0.971	0.019	0.000	0.000
LT	80.15 ± 0.99	80.00 ± 0.02	75.82 ± 15.64	56.21 ± 23.13	45.62 ± 7.57	0.982	0.488	0.000	0.000
FW-LT	80.62 ± 0.84	80.73 ± 1.70	76.24 ± 7.33	65.25 ± 5.00	41.18 ± 3.85	0.959	0.034	0.000	0.000

H&Y = Hoehn–Yahr stage, HC = healthy control, FW = forward, BW = backward, RT = right, LT = left, FW-RT = forward-right, FW = forward-left, BWRT = backward-right, BW-LT = backward-left. Data are presented as the mean ± standard deviation. Red numbers indicate significance.

## Data Availability

The datasets analyzed during the current study are available from the corresponding author on reasonable request
